# Effect of low-level laser therapy on en masse retraction in females with bimaxillary dentoalveolar protrusion: A single-center randomized clinical trial

**DOI:** 10.1007/s00056-024-00525-2

**Published:** 2024-06-06

**Authors:** Heba Mohamed Dehis, Fouad Aly El Sharaby, Faten Husain Eid, Yehya Ahmed Mostafa

**Affiliations:** 1 Orthodontic Department, Faculty of Dentistry, Cairo University, 11 El Sarayat street, El Manial—Cairo, Egypt; 2 Orthodontic Department, Faculty of Oral and Dental Medicine, Future University, Cairo, Egypt

**Keywords:** Corrective orthodontics, Orthodontic anchorage procedures, Bimaxillary protrusion, Cone-beam computed tomography, Duration of treatment, Korrigierende Kieferorthopädie, Kieferorthopädische Verankerungsverfahren, Bimaxilläre Protrusion, Digitale Volumentomographie, Behandlungsdauer

## Abstract

**Background:**

Acceleration of tooth movement has gained remarkable attention during the last decade. The aim of this study was to evaluate the effect of low-level laser therapy (LLLT) on en masse retraction of upper anterior teeth in adult women with bimaxillary dentoalveolar protrusion.

**Materials and methods:**

In this two-arm parallel trial, 36 women with bimaxillary dentoalveolar protrusion were randomly divided into two equal groups. Eligibility criteria included class I Angle molar relationship, good general and oral health as well as no systemic disease or syndrome. Four temporary anchorage devices (TADs) were used in the upper and lower arches for anchorage purposes. A 0.019×0.025-inch stainless steel wire with crimped hooks just distal to the maxillary canines was inserted. Nickle titanium (NiTi) closed coil springs (200 g/side) were employed for en masse retraction following extraction of the first premolars. In the laser group (LG), retraction of the upper anterior teeth was done along with the application of LLLT on days 0, 3, 7, and 14 after extraction and then repeated biweekly until the end of retraction. Retraction was completed without LLLT application in the nonlaser group (NLG). Data concerning the rate of retraction as well as first molars and anterior positional changes were gained from digitized models and cone beam computed tomography (CBCT) scans taken just before extraction and at the end of retraction. Treatment-associated pain and root resorption were evaluated using visual analogue scale (VAS) and CBCT scans, respectively.

**Results:**

Four patients dropped out prior to follow-up. The duration of retraction was 10.125 ± 2.876 and 13.643 ± 3.455 months in the LG and NLG, respectively. The LG showed a statistically significant faster rate of en masse retraction (0.833 ± 0.371 mm/month) compared to the NLG (0.526 ± 0.268 mm/month; *P* ≤ 0.035). The observed root resorption was significantly less in the LG (*P* ≤ 0.05) with comparable pain scores in both groups.

**Conclusions:**

Within the constraints of the parameters of the LLLT used in the current study and despite the statistically significant results on the rate of en masse retraction and the associated root resorption, LLLT did not demonstrate a clinically relevant effect that justifies its use to enhance en masse retraction.

**Name of the registry:**

Clinicaltrials.gov

**Trial registration number:**

NCT05183451

**Date of registration:**

January 10, 2022, “Retrospectively registered”

**URL of trial registry record:**

https://www.clinicaltrials.gov/study/NCT05183451

**Supplementary Information:**

The online version of this article (10.1007/s00056-024-00525-2) contains supplementary material, which is available to authorized users.

## Abstract (de)

**Hintergrund:**

Die beschleunigte Verschiebung von Zähnen ist in den letzten zehn Jahren deutlich in den Blickpunkt gerückt. Ziel dieser Studie war es, die Wirkung einer Low-Level-Lasertherapie (LLLT) auf die En-masse-Retraktion der oberen Frontzähne bei erwachsenen Frauen mit bimaxillärer dentoalveolärer Protrusion zu untersuchen.

**Materialien und Methoden:**

In dieser zweiarmigen Parallelstudie wurden 36 Frauen mit bimaxillärer dentoalveolärer Protrusion randomisiert auf zwei gleiche Gruppen aufgeteilt. Zu den Einschlusskriterien gehörten eine Angle-Klasse-I-Molaren-Beziehung, ein guter allgemeiner wie oraler Gesundheitszustand und keine systemischen Krankheiten oder Syndrome. Zur Befestigung wurden 4 provisorische Verankerungen (TADs) in den oberen und unteren Bögen verwendet. Ein 0,019×0,025-Inch-Edelstahldraht mit aufgeklemmten Häkchen wurde knapp distal der Oberkiefereckzähne eingesetzt. Geschlossene Spiralfedern aus Nickel-Titan (NiTi; 200 g/Seite) wurden für die En-masse-Retraktion nach Extraktion der ersten Prämolaren verwendet. In der Lasergruppe (LG) wurde die Retraktion der oberen Frontzähne zusammen mit der Anwendung von LLLT an den Tagen 0, 3, 7 und 14 nach der Extraktion durchgeführt und dann alle 2 Wochen bis zum Ende der Retraktion wiederholt. In der Nicht-Laser-Gruppe (NLG) wurde die Retraktion ohne LLLT-Anwendung durchgeführt. Die Daten zur Retraktionsrate sowie zu den ersten Molaren und den Positionsveränderungen im Frontzahnbereich wurden anhand von digitalisierten Modellen und DVT(digitale Volumentomographie)-Aufnahmen unmittelbar vor der Extraktion und am Ende der Retraktion gewonnen. Behandlungsbedingte Schmerzen und Wurzelresorption wurden anhand der visuellen Analogskala (VAS) bzw. der DVT-Aufnahmen bewertet.

**Ergebnisse:**

Vier Patientinnen verließen die Studie vor der Nachuntersuchung. Die Dauer der Retraktion betrug 10,125 ± 2,876 bzw. 13,643 ± 3,455 Monate in der LG bzw. in der NLG. In der LG zeigte sich eine statistisch signifikant schnellere Rate der En-masse-Retraktion (0,833 ± 0,371 mm/Monat) als in der NLG (0,526 ± 0,268 mm/Monat; *p* ≤ 0,035). Die beobachtete Wurzelresorption war in der LG signifikant geringer (*p* ≤ 0,05), die Schmerzwerte in beiden Gruppen vergleichbar.

**Schlussfolgerungen:**

Im Rahmen der Parameter der in der vorliegenden Studie verwendeten LLLT hat die LLLT trotz der statistisch signifikanten Ergebnisse hinsichtlich der Rate der En-masse-Retraktion und der damit verbundenen Wurzelresorption keine klinisch relevante Wirkung gezeigt, die ihre Anwendung zur Optimierung der En-masse-Retraktion rechtfertigt.

**Datum der Registrierung:**

10. Januar 2022, „nachträglich registriert“

## Introduction

The long duration required to achieve proper occlusion and acceptable facial esthetics continues to be a major shortcoming of orthodontic treatment. Unfortunately, this long duration is associated with increased patient discomfort and the risk of unfavorable side effects including enamel demineralization and external root resorption. Variable modalities have been implemented to enhance the rate of orthodontic tooth movement, hence, reducing the treatment duration. This was achieved either through modulating biomechanics via adopting a variable appliance design [12] and/or manipulating force delivery [31], or through modifying tissue biology via promoting paradental tissues remodeling [22].

Low-level laser therapy (LLLT) has been implemented as a noninvasive approach not only to accelerate the rate of orthodontic tooth movement but also to alleviate the associated pain and root resorption [28, 38]. Its mechanism of accelerating tooth movement was reported to be through enhancing bone remodeling via promoting the expression of osteoclastogenic factors including, among others, receptor activator of nuclear factor kappa B and its ligand (RANK/RANKL) [17] and macrophage colony-stimulating factor (M-CSF) and its receptor (c-Fms) [44]. In addition, it has been demonstrated that LLLT reduced pain perception by inhibiting the release of arachidonic acid, which decreased the levels of prostaglandin E2 [30] together with inducing the release of an endogenous opioid that produced a potent analgesic effect [21]. Moreover, a positive effect of photobiomodulation on root resorption repair has been suggested by de Melo Conti et al. via stimulating osteoprotegerin (OPG) expression prompting a decrease in the number of clastic cells at the root surface and avoiding the progression of the root resorption process [11].

Despite being intensively studied in the past few years, the application of LLLT to accelerate orthodontic tooth movement and reduce the associated pain and root resorption has shown inconclusive results [4, 9, 38].

## Specific objectives and null hypotheses

The primary objective of the current study was to evaluate the effect of LLLT on the rate of en masse retraction of upper anterior teeth in adult women with bimaxillary protrusion via consecutive digital model superimposition. The associated pain, positional changes of molars and anterior teeth as well as root resorption of anterior teeth were also evaluated as secondary outcomes using the visual analog scale (VAS) score, cone beam computed tomography (CBCT) as well as digital model scans. The null hypothesis assumed that there would be no difference in the rate of en masse retraction with or without the application of LLLT.

## Methods

### Trial design

This was a single-center, two-arm, parallel, randomized clinical trial with a 1:1 allocation ratio following the Consolidated Standards of Reporting Trials (CONSORT) statement reporting guidelines. The study method was approved by the Faculty of Dentistry Ethical Committee, Cairo University (No. 1522015). No changes to the trial execution were reported after commencement of the study.

### Participants, eligibility criteria, and settings

In all, 59 consecutive patients seeking orthodontic treatment were screened. Patients’ eligibility criteria are presented in Supplementary Table 1. Consent was obtained from all the patients before being enrolled into the study. Full pretreatment records were obtained for all participants. Fixed preadjusted Roth 0.022-inch brackets (American Orthodontics, Sheboygan, WI, USA) were bonded. Leveling and alignment proceeded till reaching 0.019×0.025-inch stainless steel (SS) arch wires. Patients were then referred for first premolar extractions and en masse retraction was immediately initiated.

### Intervention and outcomes

For every patient four temporary anchorage devices (TADs; 1.6 × 8‑mm, bracket head design; Dual Top Anchor System, Jeil Medical Corporation, Seoul, Korea) were used in the upper and lower arches (inserted buccally between the second premolars and first molars at the level of the mucogingival junction) for anchorage purposes. A fifth TAD was inserted paramedian along the line between the maxillary second premolar and the first molar to serve as a stable landmark for measurement and digital model superimposition.

Records including photographs as well as digital models and CBCT scans (Acteon X‑mind Trium CBCT machine, Merignac, France; 90 kV, 8 mA medium FOV) were taken for all participants before retraction. In accordance with the ALARA (as low as reasonably achievable) guidelines [35], a medium CBCT field of view was used. On the day of extraction, each patient was randomly assigned to either LG or NLG and received VAS pain scoring sheets. Patients were asked to fill the pain scoring sheet every day starting from the day of extraction and proceeding monthly for 6 months.

**Fig. 1:**
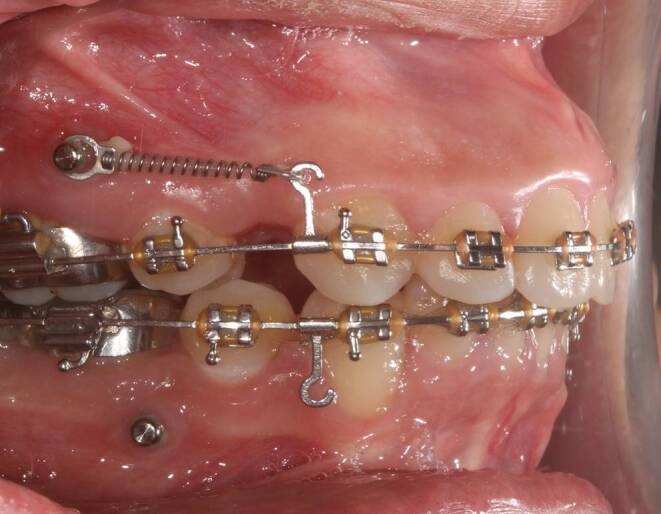
Appliance set up used for en masse retraction Apparatur für die En-masse-Retraktion

Hooks (8 mm, GNI Orthodontics, Korea) were crimped to the 0.019×0.025-inch stainless steel (SS) arch wire just distal to the maxillary canines. NiTi closed coil springs (Morelli Ortodontia, Sorocaba—SP, Brazil) were used for en masse retraction delivering a force of 200 g/side applied from the hooks to the buccal TADs directly (Fig. 1).

For the LG participants, en masse retraction was commenced with the application of LLLT (Epic™ 10 diode laser machine, BIOLASE, Foothill Ranch, CA, USA) following the parameters illustrated in Supplementary Table 2 and Supplementary Fig. 1 [19, 24, 40]. All safety and infection control measures for laser application were strictly followed. The retraction phase was considered finished when a class I canine relationship and normal overjet were achieved concurrent with improved and balanced facial profile.

### Measurements

**Fig. 2:**
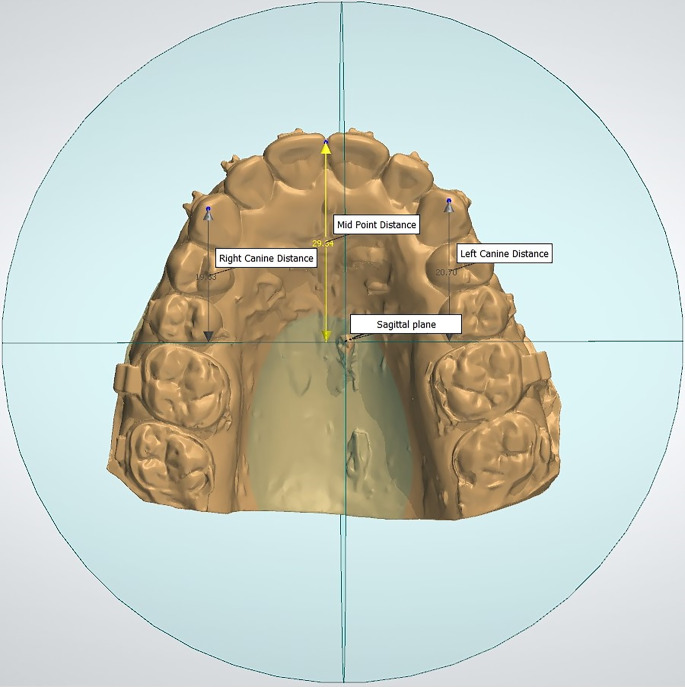
Digital model to measure en masse retraction. *MPD* Perpendicular distance between the most incisal contact point between the central incisors and coronal plane registered at the palatal miniscrew implants (MSI) Digitales Modell für die Messung der En-masse-Retraktion. *MPD* Senkrechter Abstand zwischen dem am weitesten inzisal gelegenen Kontaktpunkt zwischen den zentralen Schneidezähnen und der an den palatinalen Minischraubenimplantaten (MSI) registrierten koronalen Ebene

To accurately assess the rate of en masse retraction of the upper anterior teeth, stone models were prepared monthly and scanned using the 3Shape scanner (R500, 3Shape, Copenhagen, Denmark). Landmark identification as well as measurements were done on the digital models using the 3Shape analyzer software (3Shape, Copenhagen, Denmark; Fig. 2). The consecutive digital models were superimposed using the 3Shape analyzer software taking the most identifiable medial points on the right and left third rugae as well as the midpoint of the paramedian palatal miniscrew as a reference for superimposition. The change in the anterior segment in the superimposed models was measured to detect the rate as well as the overall amount of en masse retraction (Supplementary Fig. 2). The rate of en masse retraction was calculated from the difference in the anterior segment position in the consecutive models.

Root resorption was graded according to the Levander and Malmgren index [25] using CBCT scans [27]. CBCT sections were used to orient the cut at each anterior tooth. Reorientation was done in each slice so that the long axis of the tooth/root coincided with the vertical axis within the CBCT image (Supplementary Fig. 3). This provided optimal visualization of the tooth/root in axial, coronal, and sagittal planes [27]. Root length measurements were made along the axis of the root and graded.

Two examiners graded root resorption of the six upper anterior teeth separately and in case of any disagreement a new evaluation was made and this consensus was for the final evaluation. Measurements by one assessor were repeated at a 1 week interval to rule out intraobserver variability.

Anterior teeth tip and torque were assessed via CBCT where the long axes of the anterior teeth were constructed. The canines’ torque was assessed by measuring the long axis inclination relative to the horizontal (HP) and midsagittal (MSP) planes. Meanwhile, the upper incisors’ inclination of each tooth relative to the coronal (CP), frontal (FP), and HP planes was measured to assess the anterior teeth’s torque. Furthermore, upper incisor tip was assessed by measuring the long axes’ angulation relative to the HP and MSP, while the canine and first molar tip was assessed by angulation relative to the HP, FP, and CP.

First molar’s displacement was evaluated using the CBCT by locating its mesiobuccal cusp tip and measuring the distance to both the CP and FP from the sagittal view. The same process was repeated for the first molar’s center-point and root apex to record the whole molar displacement. On the other hand, first molar’s rotation was assessed via assessment of the scanned digital model, where the distance between the mesiopalatal cusp tip as well as the distobuccal cusp tip compared to MSP was measured (Supplementary Tables 3–5).

### Sample size calculation

The sample for this study was calculated with an alpha value of 0.05 and a power of 80% based on the study conducted by Upadhyay et al. [41], in which the mean duration of en masse retraction was 8.6 ± 2.2 months. The resultant sample size was 16 patients per group to be able to detect a minimum clinically important difference of 2.6 ± 2.5 months calculated using G power software (Universität Düsseldorf, Düsseldorf, Germany). To guard against possible attrition, 18 patients per group were taken with a total sample size of 36 patients.

### Randomization and blinding

A computer-generated random list was created with the aid of Microsoft Excel (2013, Microsoft, Redmond, WA, USA) and allocation concealment was achieved with opaque sealed envelopes. Due to the nature of the study, it was not possible to mask the patients or principal investigator. However, the outcome assessor was masked to the intervention.

Patients were randomly allocated into two groups: in the LG, patients received en masse retraction with LLLT application, while in the NLG, patients received en masse retraction without LLLT.

### Statistical analysis

Analysis was done using Statistical Package for the Social Sciences (SPSS) version 22.0 for Windows (IBM SPSS Statistics for Windows, Version 22.0. Armonk, NY). Descriptive statistics were presented as means and standard deviations for parametric numerical data. Both Cronbach alpha coefficient of association and interclass correlation coefficient (ICC) were used to assess inter- and intraobserver agreement. One-way analysis of variance (ANOVA) and Bonferroni t‑test for pairwise comparison was used to assess significant changes within each group over time and independent t‑test and Mann–Whitney U test to compare between two groups. The significance level was set at *P* ≤ 0.05.

## Results

**Fig. 3:**
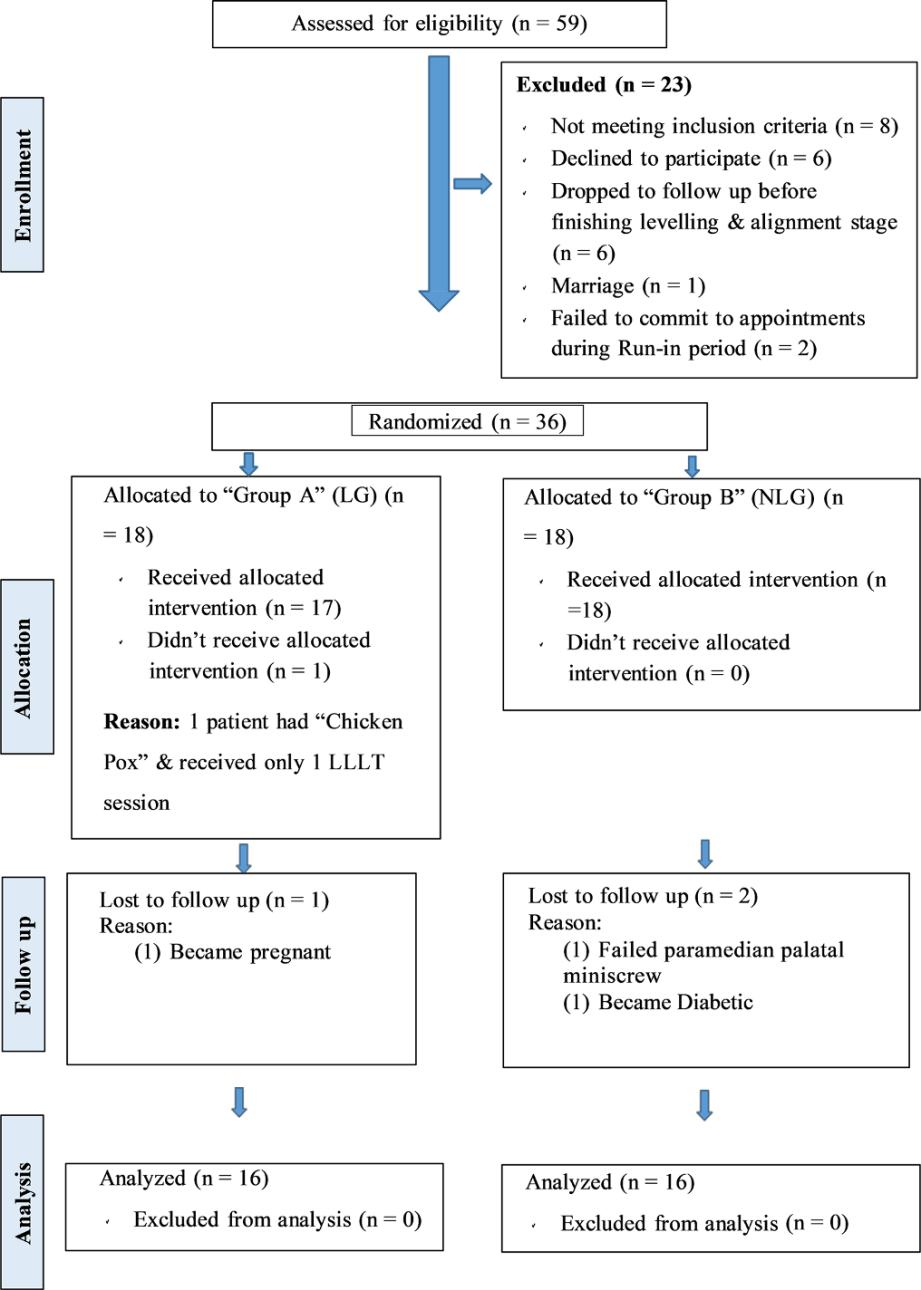
Consolidated Standards of Reporting Trials (CONSORT, 2010) participant flow diagram CONSORT (Consolidated Standards of Reporting Trials, 2010), Teilnehmendenablaufdiagramm

Of the 36 recruited patients, 4 dropped out prior to follow up (two in each group) for variable reasons mentioned in the CONSORT flow chart (Fig. 3). Hence, the power of study was maintained with a total of 32 analyzed subjects. The participants baseline skeletal and dental characteristics are presented in Supplementary Table 6. Acceptable intra- and interobserver reliability agreement between all the readings were found to be 0.992 and 0.981, respectively.

The total duration of retraction (TRDu) was statistically higher in the NLG compared to the LG (13.643 ± 3.455 and 10.125 ± 2.876 months, respectively; *P* ≤ 0.004). Moreover, a statistically significant difference was reported between both groups concerning the rate of retraction where LG showed a faster rate of retraction (0.833 ± 0.371 mm/month) compared to NLG (0.526 ± 0.268 mm/month; *P* ≤ 0.035; Table 1).

**Table 1:** Means and standard deviations (SD) for the rate of en masse retraction for both groups Mittelwerte und Standardabweichungen (SD) für die Rate der En-masse-Retraktion für beide Gruppen

	LG (*N* = 16)		NLG (*N* = 16)			t‑test			95% CI	
	Mean (mm)	±SD	Mean (mm)	±SD	Difference (mm)	t‑value	*P*-value	Effect size	Upper	Lower
1st month	0.704	0.513	0.473	0.332	0.231	0.776	0.463	0.521	1.236	−0.176
2nd month	0.922	0.606	0.337	0.351	0.585	1.701	0.131	1.152	1.927	0.419
3rd month	1.275	0.575	0.363	0.222	0.912	2.961	0.025*	2.040	2.952	1.210
4th month	1.072	0.858	0.935	0.44	0.137	0.288	0.782	0.196	0.894	−0.496
5th month	0.977	0.766	0.567	0.441	0.41	0.82	0.45	0.639	1.363	−0.062
6th month	0.475	0.0173	0.667	0.607	−0.192	−0.654	0.542	−0.436	0.259	−1.146
7th month	1.353	0.761	1.04	1.359	0.313	0.393	0.711	0.277	0.978	−0.415
8th month	1.017	1.506	0.68	0.269	0.337	0.298	0.785	0.304	1.006	−0.388
9th month	0.303	0.418	0.505	0.403	0.202	1.392	0.174	−0.480	0.216	−1.192
10th month	0.231	0.192	0.23	0.325	−0.001	−0.011	0.991	0.004	0.697	−0.689
11th month	N/A	N/A	0.47	0.438	N/A	N/A	N/A			
12th month	N/A	N/A	0.05	0.0707	N/A	N/A	N/A			
Rate of retraction	0.833	0.371	0.526	0.268	−0.307	−2.251	0.035*	0.002	0.695	−0.691
TRDu	10.125	2.876	13.643	3.455	3.518	3.13	0.004*	−1.079	−0.352	−1.846

*LG* laser group, *NLG* nonlaser group, *TRDu* total retraction duration, *95% CI* 95% confidence interval, *N/A* not applicable

*Significant when *P* ≤ 0.05

Changes in the upper first molars were evident in both groups after retraction. A negative change in the angle between the first molars and the frontal plane (constructed at the PNS) indicated a distal molars’ tip. When comparing both groups, the results revealed an increased distal molar tip and displacement in the LG than in the NLG, speaking for more anchorage gain in this group (Tables 2 and 3). By comparing the pre- and postretraction crown and root’s displacements in both groups, an overall more distal root tip was seen for both the right and left sides.

**Table 2:** Mean values of pre- and posttreatment changes of first molars for the laser group (LG) and nonlaser group (NLG) Mittelwerte der Veränderungen an den ersten Molaren vor und nach der Behandlung für die Lasergruppe (LG) und die Nichtlasergruppe (NLG)

	LG (*N* = 16)		NLG (*N* = 16)			t‑test			95% CI	
	Mean	±SD	Mean	±SD	Difference	t‑value	*P*-value	Effect size	Upper	Lower
*Left*										
UL6-C/Dis	−0.0922	1.98	−0.9	3.26	0.8028	0.657	0.519	0.292	0.994	−0.400
UL6-C/Dis2	−0.203	1.592	−0.07	1.981	−0.1307	−0.165	0.871	−0.072	0.620	−0.767
UL6MB‑C.T/Dis	0.258	3.38	−1.24	3.652	1.496	0.973	0.342	0.415	1.123	−0.279
UL6MB‑C.T/Dis2	0.148	3.102	−0.41	2.201	0.56	0.497	0.625	0.202	0.901	−0.489
UL6MB-R/Dis	−0.443	1.798	−0.55	2.94	0.109	0.0987	0.922	0.0428	0.737	−0.649
UL6MB-R/Dis2	−0.554	1.505	0.275	1.931	−0.829	−1.078	0.294	−0.467	0.228	−1.178
*Right*										
UR6-C/Dis	−1.136	2.353	−2.2	4.349	1.065	0.667	0.512	0.297	0.999	−0.395
UR6-C/Dis2	−1.248	2.475	−1.38	3.785	0.128	0.0891	0.93	0.040	0.734	−0.652
UR6MB‑C.T/Dis	−0.797	1.13	−3.67	8.523	2.873	0.998	0.33	0.461	1.172	−0.234
UR6MB‑C.T/Dis2	−0.907	1.448	−2.85	8.403	1.939	0.68	0.504	0.314	1.017	−0.378
UR6MB-R/Dis	−1.477	4.085	−0.73	2.906	−0.747	−0.503	0.621	−0.205	0.486	−0.904
UR6MB-R/Dis2	−1.588	4.125	0.094	1.123	−1.6818	−1.41	0.174	−0.542	0.155	−1.259

*95% CI* 95% confidence interval

* Significant when *P* ≤ 0.05

**Table 3:** Mean values and standard deviations (SD) of differences for the tip of anterior teeth and first molars for the laser group (LG) and nonlaser group (NLG) Mittelwerte und Standardabweichungen (SD) der Unterschiede für die Frontzahnspitze und die ersten Molaren für die Lasergruppe (LG) und die Nichtlasergruppe (NLG)

	LG (*N* = 16)		NLG (*N* = 16)			t‑test			95% CI	
	Mean	±SD	Mean	±SD	Difference	t‑value	*P*-value	Effect size	Upper	Lower
*Left*										
UL1-Tip/MSP	1.569	6.661	0.158	8.411	1.411	0.419	0.68	0.181	0.879	−0.510
UL1-Tip/HP	−1.572	6.659	−0.18	8.399	−1.395	−0.415	0.682	−0.179	0.512	−0.877
UL2-Tip/MSP	−7.569	24.091	−2.53	7.218	−5.037	−0.716	0.482	−0.276	0.416	−0.978
UL2-Tip/Hp	7.526	24.098	2.534	7.218	4.992	0.709	0.486	0.274	0.975	−0.418
UL3-Tip/CP	−7.248	6.352	−7.46	3.642	0.214	0.101	0.921	0.040	0.734	−0.652
UL3-Tip/FP	−7.401	6.351	−7.46	3.642	0.061	0.0288	0.977	0.011	0.704	−0.682
UL3-Tip/HP	7.248	6.352	7.425	3.652	−0.177	−0.083	0.935	−0.033	0.659	−0.727
UL6-Tip/CP	−1.208	4.503	0.172	4.639	−1.38	−0.694	0.496	−0.294	0.398	−0.996
UL6-Tip/FP	−1.46	4.946	0.276	4.887	−1.736	−0.815	0.424	−0.344	0.348	−1.049
UL6-Tip/HP	1.208	4.505	−0.17	4.638	1.378	0.693	0.496	0.294	0.996	−0.398
*Right*										
UR1-Tip/MSP	−1.523	2.47	0.952	4.304	−2.475	−1.55	0.137	−0.688	0.0156	−1.415
UR1-Tip/HP	1.503	2.442	−0.95	4.303	2.453	1.54	0.139	0.683	1.411	−0.019
UR2-Tip/HP	0.378	4.761	−0.4	7.104	0.773	0.284	0.779	0.125	0.821	−0.566
UR2-Tip/MSP	−0.377	4.761	0.395	7.102	−0.772	−0.284	0.78	−0.124	0.567	−0.820
UR3-Tip/CP	−3.018	6.003	−7.06	3.104	4.045	2.076	0.051	0.824	1.564	0.114
UR3-Tip/FP	−3.018	6.003	−7.39	3.628	4.37	2.133	0.045*	0.859	1.603	0.147
UR3-Tip/HP	3.018	6.003	7.065	3.105	−4.047	−2.076	0.051	−0.825	−0.115	−1.565
UR6-Tip/CP	4.2	9.057	−6.8	23.12	11.002	1.349	0.192	0.611	1.332	−0.089
UR6-Tip/FP	4.256	9.044	−7.11	23.09	11.363	1.395	0.178	0.632	1.355	−0.069
UR6-Tip/HP	−4.202	9.059	6.802	23.12	−11.004	−1.349	0.192	−0.611	0.089	−1.33

*95% CI* 95% confidence interval

* Significant when *P* ≤ 0.05

In addition, a greater palatal crown torque of the anterior teeth was significantly evident in the NLG compared to the LG (Supplementary Table 7). A mean difference in the direction of negative values denotes mesial crown tipping. This was shown to be greatest for the upper left lateral incisor and the upper left and right canines in both groups (Table 3). No significant difference between the mean difference values for both groups regarding molars rotation was evident (Supplementary Table 8).

Significantly less pain was reported in the LG compared to the NLG (*P* ≤ 0.05; Supplementary Table 9). Evaluation of the postretraction root resorption grading in both groups revealed a marked increase in the incidence of root resorption in the NLG compared to the LG which was significant for the right and left lateral incisors as well as for the left canine (Supplementary Table 10).

Throughout the study period, three buccal TADs failed with two revealing major mobility, while the third was partially dislodged. They were removed and replaced on the same day in a nearby sound area at the same height. One paramedian TAD showed mobility leading to patient exclusion from the trial.

## Discussion

“Two-step retraction” is a common technique used for extraction space closure where canines are first retracted followed by the four incisors. However, the alternative technique that involves retraction of the whole set of anterior teeth (en masse retraction) was reported to be superior with regard to the amount and time of retraction [34] as well as for anchorage preservation especially when combined with skeletal anchorage [33].

The retraction mechanics used in the current study were similar to those reported in other studies where hooks’ length ranging between 8 and 10 mm and force values between 150 and 250 g/side were implemented [3]. Contrary to the other studies, the hooks in the present trial were crimped distal to the canine to avoid possible impingement on the gingival tissue as the spring crosses over the canine eminence with maintenance of the line of action close to the center of resistance of the anterior segment.

LLLT is one of the controversial approaches that has been implemented to accelerate the rate of tooth movement [41]. The energy density of LLLT utilized in this trial was implemented based on the recommendations of Yi et al. [45] and Ge et al. [18] who reported that low energy density of 5–8 J/cm^2^ was more effective than higher ones (20 and 25 J/cm^2^ or more) for the acceleration of tooth movement. However, a multitude of laser parameters and treatment intervals have been reported by various studies [2, 15]. Hence, the recommendation of more research to elucidate the most efficient protocol was given [22]. Repeated applications of LLLT was recommended by several studies. Comparable to Doshi-Mehta et al. [14], laser application in the current study was conducted on days 0, 3, 7, and 14 in the first month and repeated biweekly until the end of retraction [23, 24, 40]. However, the literature showed variation in the protocols and timing of laser application without reporting the rationale behind any of the used protocols [14, 26, 42].

The laser whitening tip used in this study was ideal for allowing a greater area of exposure compared to the regular tip reported in other studies targeting canine rather than en masse retraction. Thus, multiple points of application per side and/or per tooth were not needed in the present study. Evasion of the use of the whitening tip in previous studies could be related to the difficulty in calculating the reached dose of laser irradiation.

In concurrence with this study’s methodology, most of the published trials applied the laser tip both buccally and palatally to the desired tooth/teeth segment, yet no standardized protocol nor justifiable location of the laser tips was previously mentioned. Some studies applied five points of laser application buccally and palatally for canine retraction [42], while others implemented three application points only [13, 46]. The main drawback of the whitening tip in the present study was the inconvenience in palatal application as the tip had to be adjusted to one target tooth at a time.

The rate of en masse retraction in the current study was calculated through superimposition of monthly prepared digital models based on stable reference points [8]. In comparable studies for en masse retraction, the rate was calculated via dividing the total amount of en masse retraction measured on lateral cephalometric radiographs over the total duration. Hence, an average rather than a precise monthly value was obtained [3]. Al-Sibaie and Hajeer [3] as well as Upadhyay et al. [41] assessed the mean treatment duration following extraction and en masse retraction, whereas other studies [6, 36] calculated the total retraction time by recording the dates from the start of retraction until the end of space closure. Yet, none of the studies assessed the actual rate of en masse retraction in a monthly manner.

On the other hand, Tuncer et al. [39] calculated the amount of space closure for each session via direct measurements in the patients’ mouth using a digital caliper and averaged it for the right and left sides. However, this method is undermined by inaccessibility of the measuring points compared to measurements implementing three-dimensional (3D) digital models which allow for better visualization and localization of the target points [42].

Three reproducible reference points were essential for the proper superimposition (tripoding) of the 3D digital models, which were fundamentally located at the most reliable and stable regions recommended by Chen et al. [8]. The paramedian palatal miniscrew served as the third stable reference point for model superimposition where its location was planned based on Silvestrini Biavati et al. [37] who recommended the palatal region for better stability of miniscrews in comparison to the buccal zones especially if placed in the area of the medial 2/3 of the third rugae and the regional palatal vault dorsal to it [8].

Application of LLLT during en masse retraction resulted in a statistically significant difference in both the total retraction duration (TRDu) as well as the rate of retraction in favor of the LG (*P* ≤ 0.035). Findings of the current study are supported by previous studies reporting a faster rate of canine retraction concomitant with LLLT application [10, 14]. Üretürk et al. [42] reported a mean increase in the rate of canine retraction at 3 months of 40% with a significant increase in the amount of canine distalization in the laser group compared to the no laser group. Likewise, Doshi-Mehta and Bhad-Patil [14] as well as Güray and Yüksel [20] detected an average increase of 30% in the rate of tooth movement for the lased groups. On the other hand, Limpanichkul et al. [26] and Mistry et al. [29] reported no significant effect of LLLT on the rate of tooth movement that was attributed to the absence of definitive clinical guidelines for the optimum protocol, dosage or frequency of LLLT.

A comparable amount of pain was felt by the patients in both groups but a greater difference in pain scores was indicated in the first month. The efficiency of LLLT in reducing pain during orthodontic treatment has been confirmed by several studies [14]; however, only very low- to low-quality evidence was reported by both Sonesson et al. [38] and Fleming et al. [16], respectively, suggesting that laser irradiation might help reducing pain during orthodontic treatment only in the short term. Moreover, following the application of two different laser energy values, AlSayed Hasan et al. [1] denied any effect of LLLT on relieving induced orthodontic pain. The differences in the observed effects of LLLT on pain perception could be related to the variations in the sample sizes together with the subjective nature of pain as well as to variation in the laser protocols adopted.

In the current trial, CBCT scans revealed minimal root resorption in association with the LG compared to the NLG. This result is concomitant with the findings of Ng et al. [32]. Ang Khaw et al. [5], on the other hand, denied any effect of LLLT on external apical root resorption (EARR), hence, confirming the questionable influence of LLLT on EARR that is acknowledged by the current evidence [28]. LLLT application was demonstrated to be effective in stimulating the remodeling activity and the regenerative capability of the connective tissues around the dental root suggesting its potential in accelerating orthodontic tooth movement, while inhibiting orthodontically induced root resorption activity. This could be attributed to an increase in the number of osteoblasts and fibroblasts in the laser treated groups [7], thus, contributing to the early repair of the resorption lacunae as well as to the synthesis and deposition of noncollagenous proteins in the cementum.

En masse retraction utilizing skeletal anchorage in both groups resulted in distal molar tip with no significant molar rotation in both groups. Interestingly, a similar distalizing effect was reported by Upadhyay et al. [41] who compared miniscrew implant anchorage to conventional anchorage during en masse retraction. Such an effect could be related to wire friction within the molar tubes resulting in a distally driving force during en masse retraction.

Xu et al. [43] reported in cases with en masse retraction a reduced incisor’s torque where extrusion and retraction of the incisal edge were obvious with no retraction at the incisor’s apex. The significant difference between both groups in the current study could be attributed to the enhanced bone modeling effect in the LG compared to the NLG group resulting in less resistance to tooth movement with subsequent more bodily.

Despite the demonstrated effect of LLLT for reducing the time needed for en masse retraction and diminishing the associated pain, the decreased treatment interval may be challenged by the number of recall visits needed for laser application. Hence, further studies are encouraged to refine the laser application protocols.

## Limitations

To date, there are no definitive clinical guidelines for the optimal dosage and frequency of LLLT to enhance the rate of tooth movement. Additional research is required to determine the ideal parameters and protocol of application and to investigate the relationship between different laser parameters and their effectiveness.

Although blinding of the participants and assessors is essential for eliminating performance bias, it was not applicable in the current study as the participants had to be notified with the nature of intervention. Blinding of both the assessor and the statistician was done to enhance validity of the results. A minimum clinically important difference (MCID) was not considered in the current study. Hence, the study might lack the necessary statistical power to identify mild to moderate effects.

## Conclusions

Despite the demonstrated statistically significant difference in the rate of en masse retraction of upper anterior teeth between the two groups, the application of low-level laser therapy (LLLT) with the parameters adopted in the current study failed to yield a clinically meaningful difference that justifies its implementation in routine clinical practice to accelerate orthodontic therapy.

## Supplementary Information

Supplementary Figures 1–3

Supplementary Tables 1–10

## Data Availability

The datasets used and/or analyzed during the current study are available from the corresponding author on reasonable request.

## Abbreviations

CBCT: Cone beam computed tomography
CP: Coronal plane
FP: Frontal plane
HP: Horizontal plane
LG: Laser group
LLLT: Low-level laser therapy
M‑CSF: Macrophage colony-stimulating factor
MSP: Midsagittal plane
NiTi: Nickle titanium
NLG: Non-laser group
OPG: Osteoprotegerin
PNS: Posterior nasal spine
RANK: Receptor activator of nuclear factor kappa B
SPSS: Statistical Package for the Social Sciences
SS: Stainless steel
TADs: Temporary anchorage devices
TRDu: Total duration of retraction
VAS: Visual analogue scale
