# Supplementary material for: Effect of low-level laser therapy on en masse retraction in females with bimaxillary dentoalveolar protrusion: A single-center randomized clinical trial
Source: J Orofac Orthop. 2024 Jun 6;86(5):284–97. doi: 10.1007/s00056-024-00525-2 (PMC12373682; doi:10.1007/s00056-024-00525-2)
Supplement: Supplementary file 1 — Supplementary Figures 1–3 [file 56_2024_525_MOESM1_ESM.pdf]

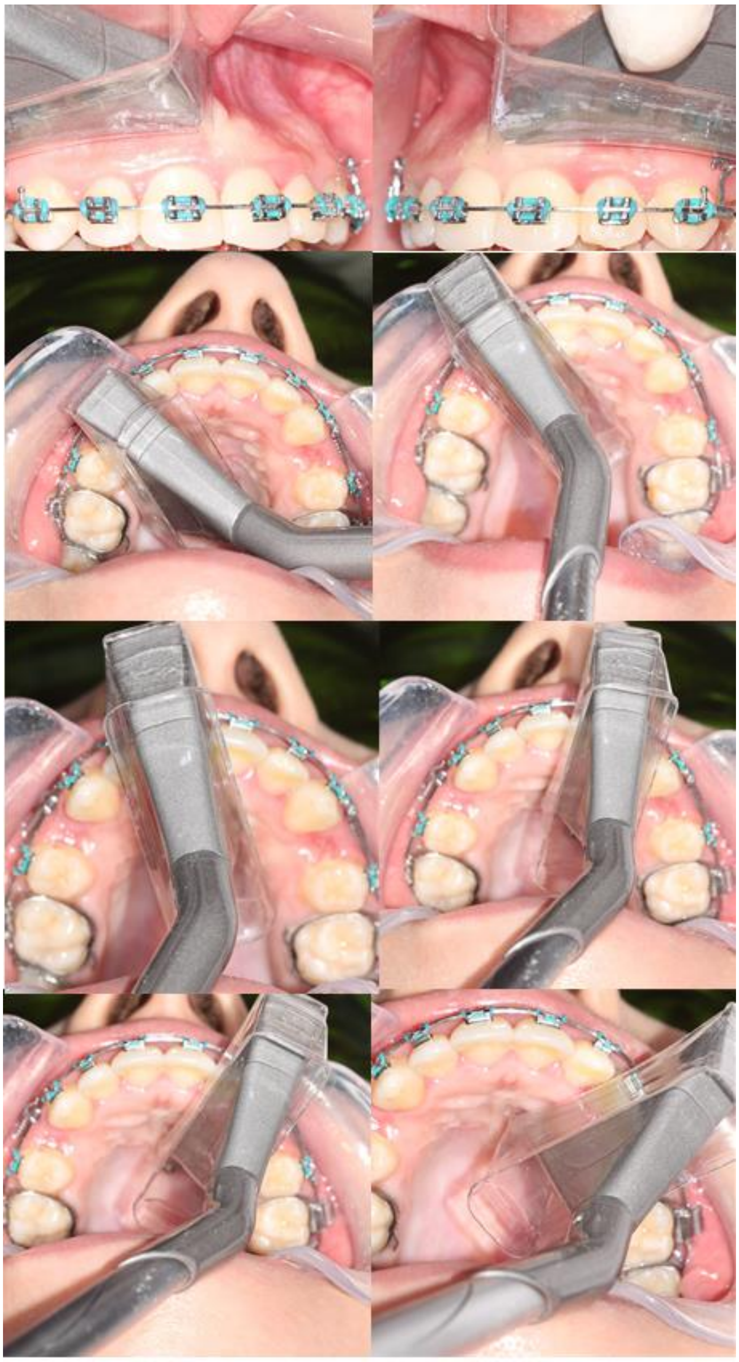

Supplementary Figure 1: Laser application zones

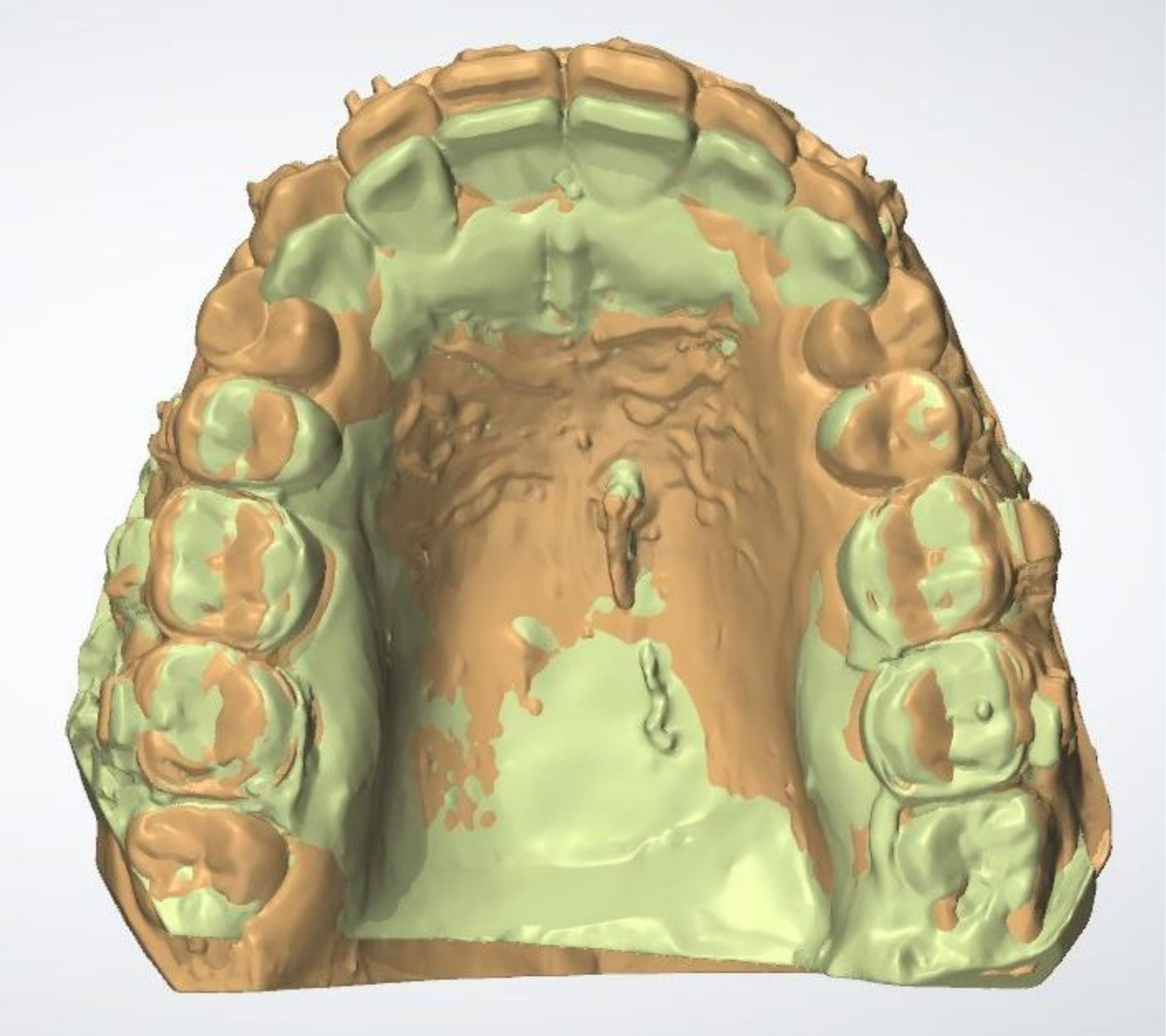

Supplementary Figure 2: Models superimposition

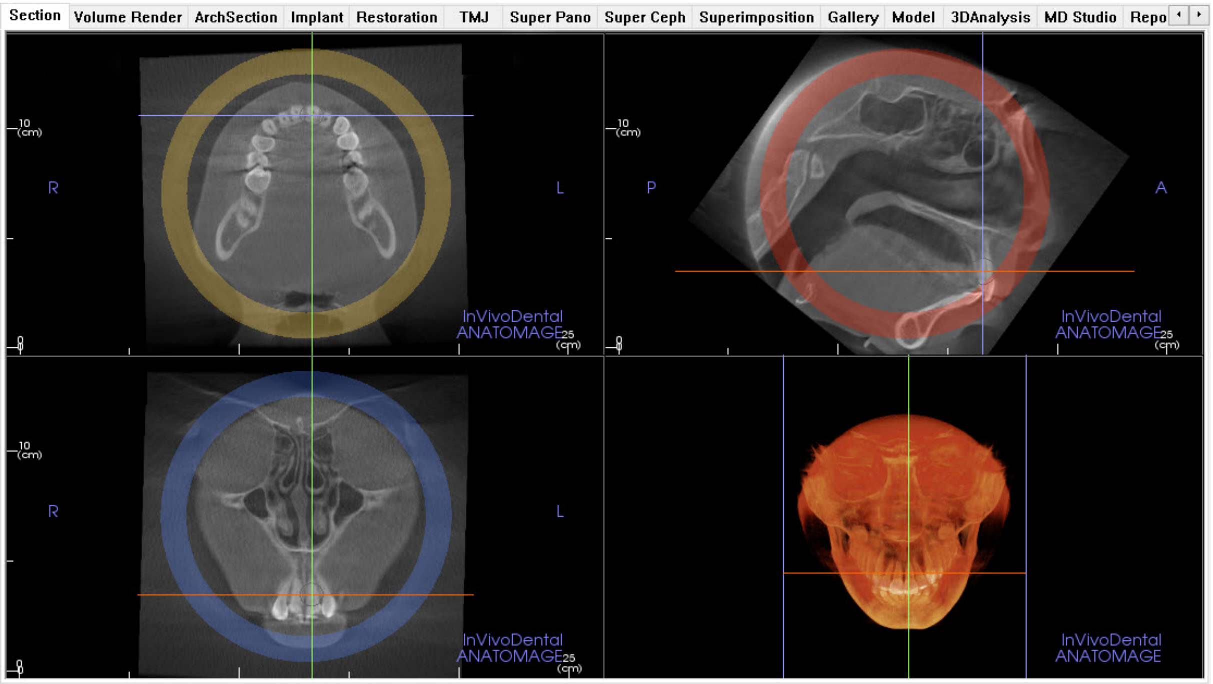

Supplementary Figure 3 A: CBCT scans (Anatomage version 5.3, Anatomage, Santa Clara, CA, USA)

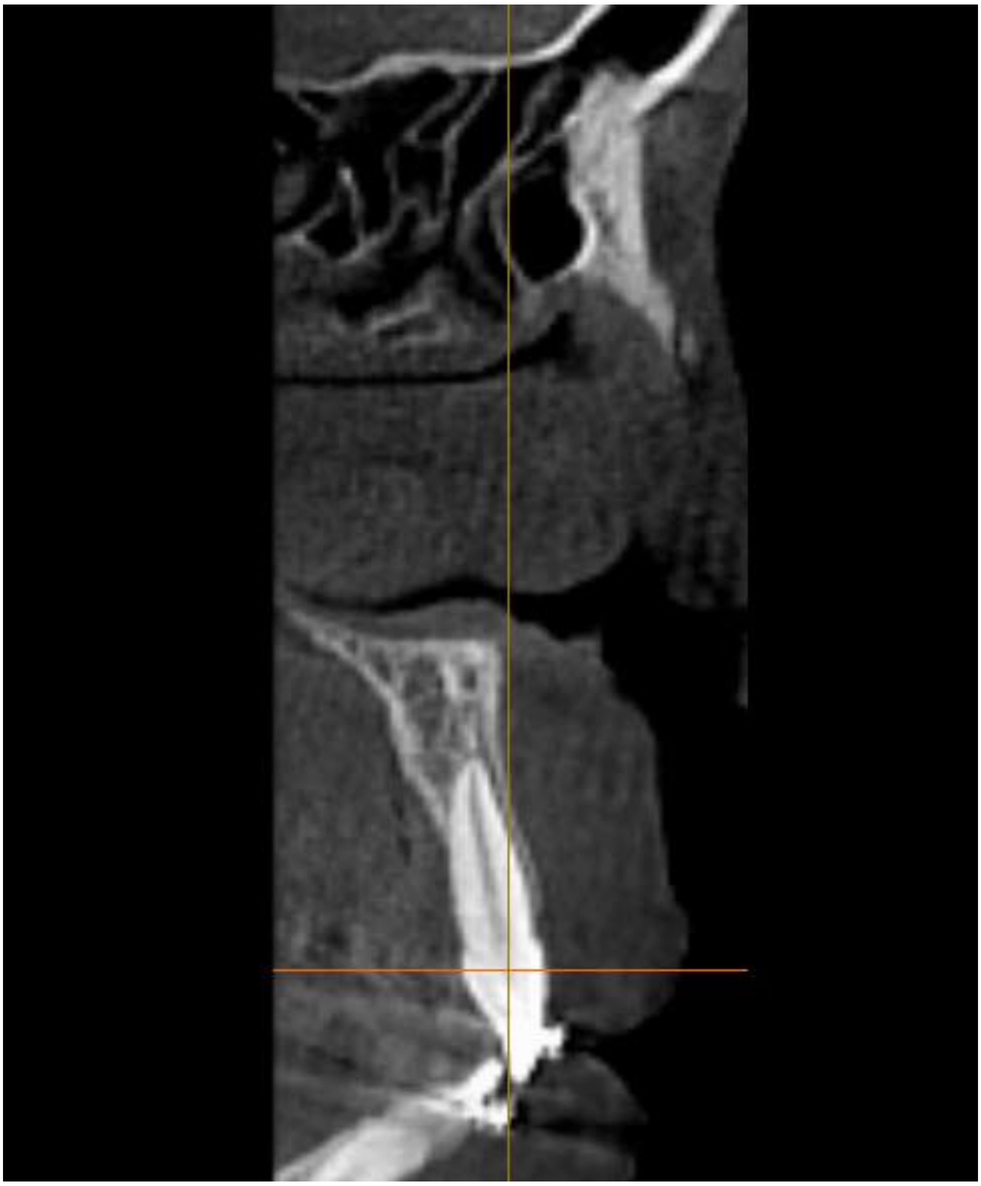

Supplementary Figure 3 B: Grade 0 (Anatomage version 5.3, Anatomage, Santa Clara, CA, USA)

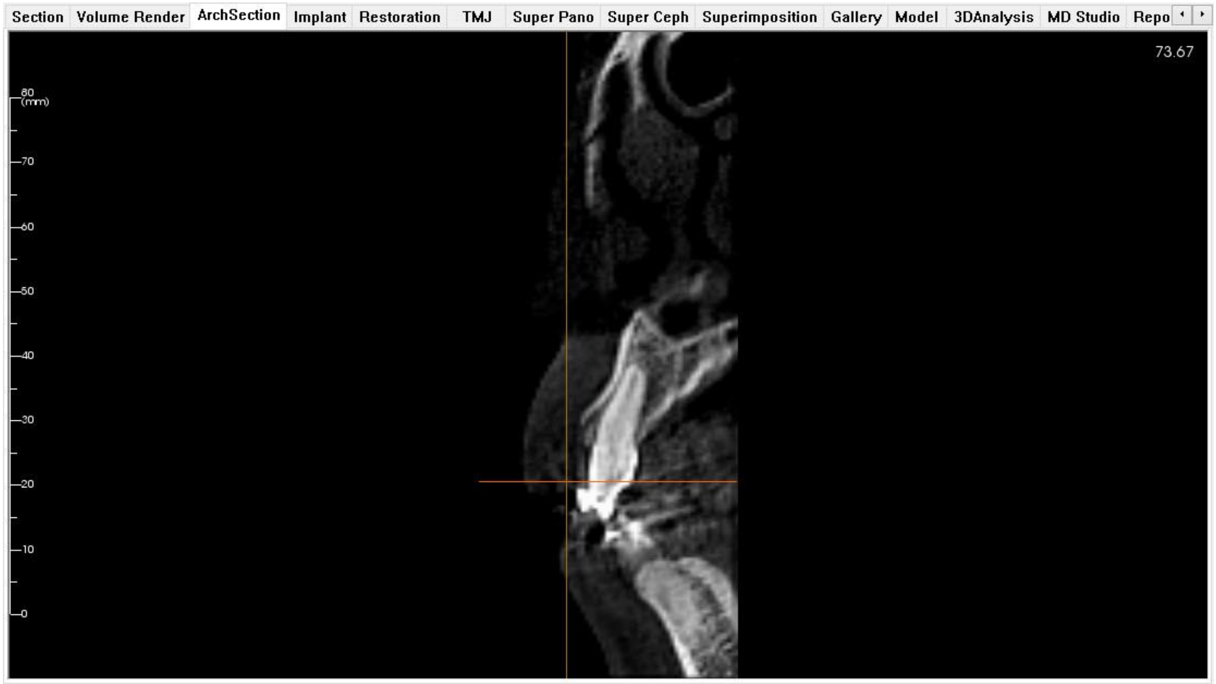

Supplementary Figure 3 C: Grade 2 (Anatomage version 5.3, Anatomage, Santa Clara, CA, USA)
