# Supplementary material for: Effect of low-level laser therapy on en masse retraction in females with bimaxillary dentoalveolar protrusion: A single-center randomized clinical trial
Source: J Orofac Orthop. 2024 Jun 6;86(5):284–97. doi: 10.1007/s00056-024-00525-2 (PMC12373682; doi:10.1007/s00056-024-00525-2)
Supplement: Supplementary file 2 — Supplementary Tables 1–10 [file 56_2024_525_MOESM2_ESM.pdf]

Supplementary Table 1: Eligibility Criteria for the Study Participants

**Inclusion criteria**	**Exclusion criteria**
• Female.<br>• Age 22± 4years<br>• Full permanent dentition (excluding 3rd molars).<br>• Good general and oral health.<br>• Class I molar relation (Angles' classification.<br>• Bimaxillary protrusion requiring extraction of four first premolars and maximum anchorage (as evident from the incisor inclination and lip profile).	• Systemic disease or syndrome.<br>• Habits that might affect treatment. (Thumb sucking or tongue thrusting)<br>• Vertical, transverse or antero-posterior skeletal discrepancies.<br>• Previous orthodontic treatment.<br>• Medications that could interfere with tooth movement.

Supplementary Table 2: Laser parameters and application zones

Medium	InGaAsP Semi-conductor diode
Power Mode	Continuous
Wavelength	940 ± 10 nm
Energy density	7.14 J/cm^2
Power output	50 mW
Application tool	Tooth whitening handpiece (35mm x 8mm)
Application zones & time	*Buccally*: at the vestibule after dividing the anterior segment into two halves (right and left) each for 60 seconds.<br>*Palatally*: at each anterior tooth for 40 seconds
Laser treatment intervals	At day of extraction, then days 7,14 ,21 ,28, 42, 56 and then repeated biweekly till the end of retraction.

Supplementary Table 3: CBCT landmarks' definitions

Landmark	Definition
Skeletal landmarks	
**ANS**	(Anterior nasal spine)<br>Most anterior point on the tip of the anterior nasal spine.
**PNS**	(Posterior nasal spine)<br>Most posterior point on the hard palate at the tip of the post nasal spine.
**PR, PL**	(Posterior maxillary point)<br>The right and left point of maximum concavity of the posterior border of the palatine bone in the horizontal plane from coronal view.
Artificial landmarks	
**P-MS**	(Paramedian palatal mini-screw)<br>Mid-point of the head of the paramedian palatal mini-screw.
Dental landmarks	
**UR3-C.T**	(Maxillary right canine cusp tip)<br>The most incisal point on the cusp tip of the maxillary right canine.
**UR3-R**	(Maxillary right canine root apex)<br>The most apical point on the root apex of the maxillary right canine.
**UR3-C**	(Maxillary right canine center point)<br>The mid-point between the UR3-C.T and UR3-R on the maxillary right canine long axis.
**UR2-I**	(Maxillary right lateral incisor's incisal edge)<br>Mid-point of the incisal edge of the maxillary right lateral incisor.
**UR2- R**	(Maxillary right lateral incisor's root apex)<br>The most apical point on the root apex of the maxillary right lateral incisor.
**UR2-C**	(Maxillary right lateral incisor's center point)<br>The mid-point between the UR2-I and the UR2-R along the long axis of the maxillary right lateral incisor.
**UR1-I**	(Maxillary right central incisor's incisal edge)<br>Mid-point of the incisal edge of the maxillary right central incisor.
**UR1-R**	(Maxillary right central incisor's root apex)<br>The most apical point on the root apex of the maxillary right central incisor.
**UR1-C**	(Maxillary right central incisor's center point)<br>The mid-point between the UR1-I and the UR1-R along the long axis of the maxillary right central incisor.
**UL1-I**	(Maxillary left central incisor's incisal edge)<br>Mid-point of the incisal edge of the maxillary left central incisor.
**UL1-R**	(Maxillary left central incisor's root apex)<br>The most apical point on the root apex of the maxillary left central incisor.
**UL1-C**	(Maxillary left central incisor's center point)<br>The mid-point between the UL1-I and the UL1-R along the long axis of the maxillary left central incisor.
**UL2-I**	(Maxillary left lateral incisor's incisal edge)<br>Mid-point of the incisal edge of the maxillary left lateral incisor.
**UL2-R**	(Maxillary left lateral incisor's root apex)<br>The most apical point on the root apex of the maxillary left lateral incisor.
**UL2-C**	(Maxillary left lateral incisor's center point)<br>The mid-point between the UL2-I and the UL2-R along the long axis of the maxillary left lateral incisor.
**UL3-C.T**	(Maxillary left canine cusp tip)<br>The most incisal point on the cusp tip of the maxillary left canine.
**UL3-R**	(Maxillary left canine root apex)

	The most apical point on the root apex of the maxillary left canine.
**UL3-C**	(Maxillary left canine center point)<br>The mid-point between the UR3-C.T and UR3-R on the maxillary left canine long axis.
**U1.1/C**	(Contact point between maxillary right and left central incisors)<br>Contact point between maxillary right and left central incisors.
**U1.1/R**	(Mid-point between root apices of maxillary central incisors)<br>Horizontal mid-point between the maxillary central incisors root apices.
**UR6MB-C.T**	(Maxillary right first molar mesio-buccal cusp tip)<br>Most occlusal point at the mesio-buccal cusp tip of the maxillary right first molar.
**UR6MB-R**	(Maxillary right first molar mesio-buccal root apex)<br>Most apical point at the root apex of the maxillary right first molar.
**UR6-C**	(Maxillary right first molar center point)<br>Mid-point between the UR6MB-C.T and the UR6MB-R along the long axis of the maxillary first molar.
**UL6MB-C.T**	(Maxillary left first molar mesio-buccal cusp tip)<br>Most occlusal point at the mesio-buccal cusp tip of the maxillary right first molar.
**UL6MB-R**	(Maxillary left first molar mesio-buccal root apex)<br>Most apical point at the root apex of the maxillary left first molar.
**UL6-C**	(Maxillary left first molar center point)<br>Mid-point between the UL6MB-C.T and the UL6MB-R along the long axis of the maxillary first molar.

Supplementary Table 4: CBCT reference planes and lines definitions

Reference planes	Definition
**HP**	(Horizontal plane)<br>Plane formed between the R-Po, L-Po and R-Or.
**MSP**	(Mid sagittal plane)<br>Plane formed between the ANS, PNS perpendicular to HP.
**CP**	(Coronal Plane)<br>Plane formed at the P-MS perpendicular to HP and MSP.
**FP**	(Frontal Plane)<br>Plane formed at the PNS perpendicular to HP and MSP.
**UR3-LA**	Line connecting UR3-C.T and UR3-R.
**UR2-LA**	(Maxillary right lateral incisor long axis)<br>Line connecting UR2-I and UR2-R.
**UR1- LA**	(Maxillary right central incisor long axis)<br>Line connecting UR1-I and UR1-R.
**UL1-LA**	(Maxillary left central incisor long axis)<br>Line connecting UL1-I and UL1-R.
**UL2-LA**	(Maxillary left lateral incisor long axis)<br>Line connecting UL2-I and UL2-R.
**UL3-LA**	(Maxillary left canine long axis)<br>Line connecting UL3-C.T and UL3-R.
**UR6-LA**	(Maxillary right first molar long axis)<br>Line connecting UR6MB-C.T and UR6MB-R
**UL6-LA**	(Maxillary left first molar long axis)<br>Line connecting UL6MB-C.T and UL6MB-R

Supplementary Table 5: CBCT measurements

Measurements	Definition
1-Total amount of anterior teeth retraction	
**U1.1/C-CP**	(Distance moved by the median contact point of maxillary central incisors)<br>Perpendicular distance from U1.1/C to Coronal Plane from sagittal view.
**U1.1/C-FP**	(Distance moved by the median contact point of maxillary central incisors)<br>Perpendicular distance from U1.1/C to Frontal Plane from sagittal view.
**U1.1/R-CP**	(Distance moved by the mid-point of root apices of maxillary central incisors)<br>Perpendicular distance from U1.1/R to Coronal Plane from sagittal view.
**U1.1/R-CP**	(Distance moved by the mid-point of root apices of maxillary central incisors)<br>Perpendicular distance from U1.1/R to Frontal Plane from sagittal view.
2-a. Distance moved by posterior teeth (Mesially or distally)	
**UR6MB-C.T/Dis**	(Maxillary right first molar mesio-buccal cusp tip displacement)<br>Distance between maxillary right first molar mesio-buccal cusp tip and Coronal Plane from sagittal view.
**UR6MB-C.T/Dis** 2	(Maxillary right first molar mesio-buccal cusp tip displacement)<br>Distance between maxillary right first molar mesio-buccal cusp tip and Frontal Plane from sagittal view.
**UR6-C/Dis**	(Maxillary right first molar center-point displacement)<br>Distance between maxillary right first molar center-point and Coronal Plane from sagittal view.
**UR6-C/Dis 2**	(Maxillary right first molar center-point displacement)<br>Distance between maxillary right first molar center-point and Frontal Plane from sagittal view.
**UR6MB-R/Dis**	(Maxillary right first molar mesio-buccal root apex displacement)<br>Distance between maxillary right first molar mesio-buccal root apex and Coronal Plane from sagittal view.
**UR6MB-R/Dis 2**	(Maxillary right first molar mesio-buccal root apex displacement)<br>Distance between maxillary right first molar mesio-buccal root apex and Frontal Plane from sagittal view
**UL6MB-C.T/Dis**	(Maxillary left first molar mesio-buccal cusp tip displacement)<br>Distance between maxillary left first molar mesio-buccal cusp tip and Coronal Plane from sagittal view.
**UL6MB-C.T/Dis 2**	(Maxillary left first molar mesio-buccal cusp tip displacement)<br>Distance between maxillary left first molar mesio-buccal cusp tip and Frontal Plane from sagittal view.
**UL6-C/Dis**	(Maxillary left first molar center-point displacement)<br>Distance between maxillary left first molar center-point and Coronal Plane from sagittal view.
**UL6-C/Dis 2**	(Maxillary left first molar center-point displacement)

	Distance between maxillary left first molar center-point and Frontal Plane from sagittal view.
**UL6MB-R/Dis**	(Maxillary left first molar mesio-buccal root apex displacement)<br>Distance between maxillary left first molar mesio-buccal root apex and Coronal Plane from sagittal view.
**UL6MB-R/Dis 2**	(Maxillary left first molar mesio-buccal root apex displacement)<br>Distance between maxillary left first molar mesio-buccal root apex and Frontal Plane from sagittal view.
2-b. Tipping of posterior teeth	
**UR6-Tip/CP**	(Maxillary right first molar tipping to Coronal Plane)<br>Angle between maxillary right first molar long axis and Coronal Plane from sagittal view.
**UR6-Tip/FP**	(Maxillary right first molar tipping to Frontal Plane)<br>Angle between maxillary right first molar long axis and Frontal Plane from sagittal view.
**UR6-Tip/HP**	(Maxillary right first molar tipping to horizontal plane)<br>Angle between maxillary right first molar long axis and Horizontal Plane from sagittal view.
**UL6-Tip/CP**	(Maxillary left first molar tipping to Coronal Plane)<br>Angle between maxillary left first molar long axis and Coronal Plane from sagittal view.
**UL6-Tip/FP**	(Maxillary left first molar tipping to Frontal Plane)<br>Angle between maxillary left first molar long axis and Frontal Plane from sagittal view.
**UL6-Tip/HP**	(Maxillary left first molar tipping to horizontal plane)<br>Angle between maxillary left first molar long axis and Horizontal Plane from sagittal view.
3-Tipping of anterior teeth	
**UR3-Tip/CP**	(Maxillary right canine tip to Coronal Plane)<br>Angle between maxillary right canine long axis and Coronal Plane from sagittal view.
**UR3-Tip/FP**	(Maxillary right canine tip to Frontal Plane)<br>Angle between maxillary right canine long axis and Frontal Plane from sagittal view.
**UR3-Tip/HP**	(Maxillary right canine tipping to horizontal plane)<br>Angle between maxillary right canine long axis and Horizontal Plane from sagittal view.
**UR2-Tip/MSP**	(Maxillary right lateral incisor tip to Coronal Plane)<br>Angle between maxillary right lateral incisor long axis and Mid-sagittal Plane from coronal view.
**UR2-Tip/HP**	(Maxillary right lateral incisor tip to horizontal plane)<br>Angle between maxillary right lateral incisor long axis and Horizontal Plane from coronal view.
**UR1-Tip/ MSP**	(Maxillary right central incisor tip to Coronal Plane)<br>Angle between maxillary right central incisor long axis and Mid-sagittal Plane from coronal view.

**UR1-Tip/HP**	(Maxillary right central incisor tip to horizontal plane)<br>Angle between maxillary right central incisor long axis and Horizontal Plane from coronal view.
**UL1-Tip/ MSP**	(Maxillary left central incisor tip to Coronal Plane)<br>Angle between maxillary left central incisor long axis and Mid-sagittal Plane from coronal view.
**UL1-Tip/HP**	(Maxillary left central incisor tip to horizontal plane)<br>Angle between maxillary left central incisor long axis and Horizontal Plane from coronal view.
**UL2-Tip/ MSP**	(Maxillary left lateral incisor tip to Coronal Plane)<br>Angle between maxillary left lateral incisor long axis and Mid-sagittal Plane from coronal view.
**UL2-Tip/HP**	(Maxillary left lateral incisor tip to horizontal plane)<br>Angle between maxillary left lateral incisor long axis and Horizontal Plane from coronal view.
**UL3-Tip/CP**	(Maxillary left canine tip to Coronal Plane)<br>Angle between maxillary left canine long axis and Coronal Plane from sagittal view.
**UL3-Tip/FP**	(Maxillary left canine tip to Frontal Plane)<br>Angle between maxillary left canine long axis and Frontal Plane from sagittal view.
**UL3-Tip/HP**	(Maxillary left canine tipping to horizontal plane)<br>Angle between maxillary left canine long axis and Horizontal Plane from sagittal view.
4-Torque of anterior teeth	
**UR3-Torque/MSP**	(Maxillary right canine torque to mid sagittal plane)<br>Angle between maxillary right canine long axis and mid-sagittal plane from coronal view.
**UR3-Torque/HP**	(Maxillary right canine torque to Horizontal plane)<br>Angle between maxillary right canine long axis and Horizontal plane from coronal view.
**UR2-Torque/CP**	(Maxillary right lateral incisor torque to Coronal Plane)<br>Angle between maxillary right lateral incisor long axis and Coronal Plane from sagittal view.
**UR2-Torque/FP**	(Maxillary right lateral incisor torque to Frontal Plane)<br>Angle between maxillary right central incisor long axis and Frontal Plane from sagittal view.
**UR2-Torque/HP**	(Maxillary right lateral incisor torque to horizontal plane)<br>Angle between maxillary right lateral incisor long axis and Horizontal plane from sagittal view.
**UR1-Torque/CP**	(Maxillary right central incisor torque to Coronal Plane)<br>Angle between maxillary right central incisor long axis and Coronal Plane from sagittal view.
**UR1-Torque/HP**	(Maxillary right central incisor torque to horizontal plane)<br>Angle between maxillary right central incisor long axis and Horizontal plane from sagittal view.
**UL1-Torque/CP**	(Maxillary left central incisor torque to Coronal Plane)<br>Angle between maxillary left central incisor long axis and Coronal Plane from sagittal view.
**UL1-Torque/HP**	(Maxillary left central incisor torque to horizontal plane)<br>Angle between maxillary left central incisor long axis and Horizontal plane from sagittal view.
**UL1-Torque/FP**	(Maxillary left central incisor torque to Frontal Plane)<br>Angle between maxillary left central incisor long axis and Frontal Plane from sagittal view.
**UL2-Torque/CP**	(Maxillary left lateral incisor torque to horizontal plane)

	Angle between maxillary left lateral incisor long axis and Horizontal plane from sagittal view.
**UL2-Torque/HP**	(Maxillary left lateral incisor torque to Coronal Plane)<br>Angle between maxillary left lateral incisor long axis and Coronal Plane from sagittal view.
**UL2-Torque/FP**	(Maxillary left lateral incisor torque to Frontal plane)<br>Angle between maxillary left lateral incisor long axis and Frontal plane from sagittal view.
**UL3-Torque/MSP**	(Maxillary left canine torque to mid sagittal plane)<br>Angle between maxillary left canine long axis and mid-sagittal plane from coronal view.
**UL3-Torque/HP**	(Maxillary left canine torque to mid sagittal plane)<br>Angle between maxillary left canine long axis and Horizontal plane from coronal view.

Supplementary Table 6: Baseline skeletal and dental characteristics of subjects in both study groups

	LG (N=16)		NLG (N=16)		P-Value
	**Mean**	**SD**	**Mean**	**SD**	
**SNA°**	82.94	1.08	83.15	1.33	0.54
**SNB °**	79.53	1.28	79.42	1.19	0.42
**ANB °**	3.41	0.85	3.73	0.87	0.76
**SN/MP°**	35.15	1.15	35.23	1.23	0.4
**FMA°**	25.04	1.08	25.38	1.54	0.64
**U1/NA (mm)**	6.75	1.46	5.72	1.22	0.97
**U1/SN°**	110.46	2.27	109.68	2.15	0.75
**L1/NB (mm)**	6.18	2.05	6.63	1.46	0.64
**L1/MP°**	99.38	2.84	101.98	2.51	0.99
**E-Plane (mm)**	5.08	0.81	5.42	0.93	0.79

* Significant when $P \leq 0.05$

Supplementary Table 7: The mean values of anterior teeth torque pre-and post-treatment in LG and NLG

	LG (N= 16)				NLG (N= 16)			
	Pre		Post		Pre		Post	
	Mean	SD	Mean	SD	Mean	SD	Mean	SD
				Left				
UL1-Torque/CP	24.56	5.334	21.527	7.534	25.413	8.092	16.471	5.521
UL1-Torque/HP	65.44	5.334	68.473	7.534	64.587	8.092	73.528	5.52
UL2-Torque/CP	23.001	8.35	18.202	5.274	24.562	7.292	14.904	6.106
UL2-Torque/FP	23.001	8.35	18.202	5.274	24.562	7.292	14.793	6.378
UL2-Torque/HP	66.999	8.35	71.798	5.274	65.438	7.292	75.096	6.106
UL3-Torque/HP	80.971	4.269	82.561	2.312	78.124	7.694	85.051	3.767
UL3-Torque/MSP	9.029	4.269	7.439	2.312	11.876	7.694	4.913	3.815
				Right				
UR1-Torque/CP	23.412	5.584	19.572	4.652	24.867	7.612	15.4	5.095
UR1-Torque/FP	23.412	5.584	19.572	4.652	24.867	7.612	15.4	5.095
UR1-Torque/HP	66.588	5.584	70.428	4.652	65.134	7.613	74.6	5.095
UR2-Torque/CP	21.907	6.171	19.484	3.552	22.235	5.044	12.193	5.354
UR2-Torque/FP	21.907	6.171	19.484	3.552	22.235	5.044	12.085	5.597
UR2-Torque/HP	68.093	6.171	70.516	3.552	67.765	5.044	77.806	5.354
UR3-Torque/HP	77.888	6.003	81.003	4.66	81.911	5.909	84.083	4.678
UR3-Torque/MSP	12.112	6.003	8.998	4.658	8.071	5.935	5.908	4.69

LG: laser group, NLG: non-laser group, *: Significant when P ≤ 0.05

Supplementary Table 8: The mean values and SD for molar rotation for LG and NLG

	LG (N= 16)		NLG (N= 16)				
	Mean	SD	Mean	SD	Difference	t-value	P- value
**Rt MP/MSP**	0.113	0.0956	0.0858	0.116	0.0268	0.611	0.548
**Rt DB/MSP**	0.0987	0.123	0.0802	0.109	0.0185	0.391	0.699
**Lt MP/MSP**	0.0671	0.0747	0.0787	0.111	-0.012	-0.294	0.771
**Lt DB/MSP**	0.0698	0.0765	0.0475	0.0717	0.0223	0.734	0.47

LG: laser group, NLG: non-laser group, *: Significant when P ≤ 0.05

Supplementary Table 9: Mean and standard deviation values for the VAS pain scores along the follow up for both groups

	LG (N= 16)		NLG (N= 16)				
	**Mean (mm)**	**SD**	**Mean (mm)**	**SD**	**Difference (mm)**	**t-value**	**P-value**
**1st month**	0.493	0.914	0.888	1.729	-0.395	-0.942	0.351
**2nd month**	0.279	0.594	0.473	1.19	-0.194	-0.679	0.5
**3rd month**	0.207	0.812	0.397	1.324	-0.19	-0.0298	0.976
**4th month**	0.164	1.39	0.225	0.492	-0.061	-0.562	0.476
**5th month**	0.12	1.241	0.197	1.227	-0.077	-0.87	0.245
**6 th month**	0.1	0.896	0.143	1.893	-0.043	-0.524	0.860

LG: laser group, NLG: non-laser group, *: Significant when $P \leq 0.05$

Supplementary Table 10: Root resorption scores for both groups

			Median	25%	75%	U Statistic	p-value
LG	UR 3	Pre	0	0	0.75	21	0.71
		Post	0	0	0		
	UR 2	Pre	0	0	0	10.5	0.073
		Post	1	0.25	1		
	UR 1	pre	0	0	1	21	0.71
		Post	1	0	1		
	UL 1	pre	0	0	1	16	0.534
		Post	1	0	1		
	UL 2	pre	0	0	0.75	21	0.71
		Post	0	0	1		
	UL 3	pre	0	0	0	21	0.71
		Post	0	0	0.75		
NLG	UR 3	Pre	0	0	0	71.5	0.305
		Post	0	0	0.25		
	UR 2	Pre	0	0	0.25	32.5	0.002*
		Post	1	1	1		
	UR 1	pre	0	0	1	65	0.255
		Post	1	0	1		
	UL 1	pre	0	0	0	64	0.321
		Post	0	0	1		
	UL 2	pre	0	0	0.25	44	0.034*
		Post	1	0	1		
	UL 3	pre	0	0	0	51	0.042*
		Post	1	0	1		

LG: laser group, NLG: non-laser group, *: Significant when $P \leq 0.05$
